# A floating electronic tag and LoRa-based infrastructure for jellyfish tracking and recapture

**DOI:** 10.1016/j.ohx.2026.e00854

**Published:** 2026-11-03

**Authors:** Quentin Ponzo, Andrea Sauviat, Delphine Bonnet, Vincent Kerzerho

**Affiliations:** a LIRMM, Univ Montpellier, CNRS, 161 rue Ada 34095 Montpellier cedex 5, France; b MARBEC, Univ Montpellier, CNRS, IFREMER, IRD, Place Eugène Bataillon - bât 24 - CC093 34095 MONTPELLIER Cedex 5, France

**Keywords:** Biologging, Jellyfish, LoRa communication, GNSS tracking, Animal telemetry, Marine ecology

## Abstract

Jellyfish biologging remains challenging due to the animals’ fragile morphology, weak buoyancy, and the absence of dedicated tracking devices. This work presents the Jellyfish float tracker, an open-source floating electronic tag and associated data infrastructure designed for jellyfish tracking and recovery. The system combines a lightweight 3D-printed waterproof float, a low-power GNSS receiver, a LoRaWAN communication transceiver, and an end-to-end data processing pipeline based on The Things Network, MQTT, Node-RED, and InfluxDB. The tag periodically acquires and transmits surface positions while storing GNSS data locally. The position transmission enables to recover the animal and the tag, while the position storage in the tag memory is used to avoid data loss due to temporary unavailable radio coverage. All hardware design files, firmware, and software resources are released under open-source licenses to enable reproduction and customization. The complete platform was validated during field deployments in a coastal lagoon, where up to 12 tags were operated simultaneously through a dedicated LoRaWAN infrastructure. Deployment durations ranging from 1 h to 96 h were demonstrated, enabling live monitoring, trajectory reconstruction, and tagged animal recovery. This open hardware platform provides a reusable solution for jellyfish biologging applications and can be adapted to other lightweight floating animal-tracking scenarios.

## Graphical abstract

**Figure:**
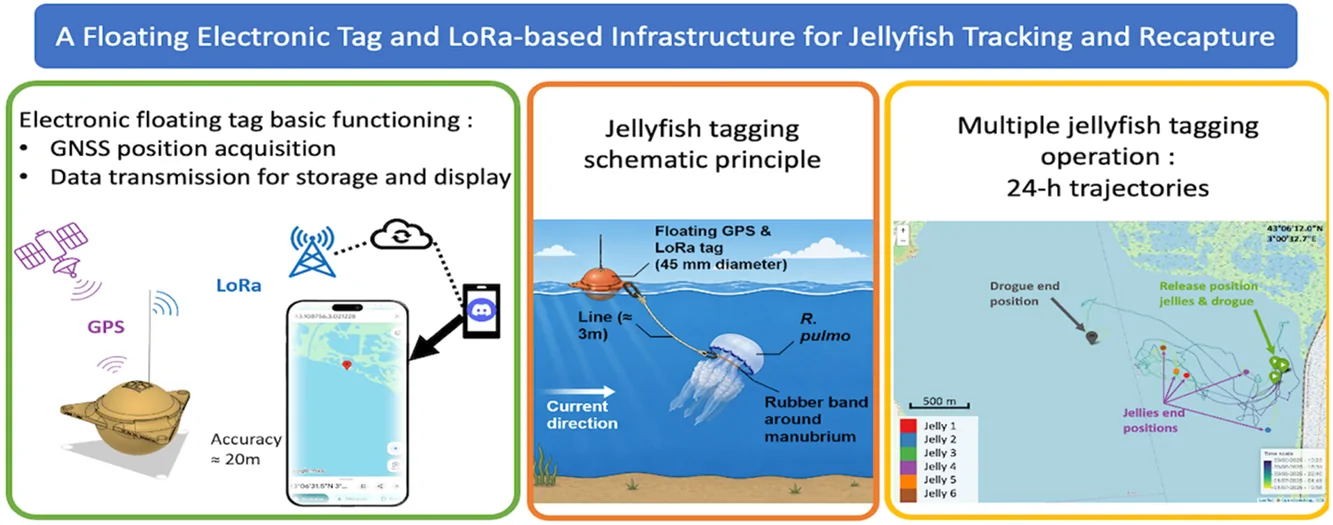

**Table:** 

**Specifications table**	

**Hardware name**	Jellyfish float tracker

**Subject area**	•Engineering and material science • Environmental, planetary and agricultural sciences

**Hardware type**	•Field measurements and sensors • Electrical engineering and computer science

**Closest commercial analog**	No direct commercial analog is available. The closest systems are proprietary wildlife or asset trackers, but they do not provide an open, jellyfish-compatible floating design together with a reproducible LoRaWAN data reception chain.

**Open source license**	CERN-OHL-S-2.0 for mechanical CAD and PCB design, MIT for firmware and software, CC-by-sa-4.0 for documentations

**Cost of hardware**	Total cost for setup = 1233.32 €, electronic tag = 136.73 €, battery charger = 5.59 €, hardware for data collection, storage and display (gateway and server) = 1091 €.

**Source file repository**	The complete source files corresponding to version 1.0.0 of the Jellyfish Float Tracker are archived on Zenodo: https://doi.org/10.5281/zenodo.21811841. The active development repository is available at: https://gite.lirmm.fr/qponzo/jellyfish-float-tracker.

## 1 Hardware in context

Jellyfish has some negative economic impacts, especially for fisheries and tourism. Regarding fishing, jellyfish is a predator of fish larvae and is in competition with fish for food. This can lead to a reduction of fish population [1]. In addition, jellyfish blooms driving to massive aggregations affect daily operations by notably filling and damaging fishing nets. Concerning tourism, jellyfish blooms prevent people from recreational water activities affecting destination choice [2], [3]. Global warming and ecosystem eutrophication have caused an increase in jellyfish populations [4], which has enlightened the need to better understand the dynamics and behavior processes of jellyfish.

A solution for animals behavior studies is biologging. Biologging technology (also known as animal telemetry, biotelemetry, or animal-borne sensors) allows researchers to collect data from free-ranging animals while they move in their environment using an electronic device that is attached to the animal and integrates some sensors and resources for storage or data transmission [5]. Biologging is used for a wide range of species, from fish [6] to birds [7], mammals [8] and bats [9]. Biologging for jellyfish has not been as much developed as for other species. There are several reasons for this limited development. First of all, jellyfish has slightly negative buoyancy and is made of soft tissue. It results in strong challenges to develop tags that have reliable attachments that does not affect the animal behavior and welfare. In addition, as jellyfish is a species neither protected nor exploited, it has benefited from less research efforts than some other ones. For all these reasons, it does not exist any biologger developed for jellyfish application. And the biologger deployments on jellyfish are limited in numbers and duration [10].

Despite biologging has benefited from Do-It-Yourself (DIY) works and open source technologies, it rarely exists fully opened biologgers including hardware, packaging, software for data transmission. For instance, [11], [12] provides open hardware electronic devices used as biologgers respectively for Eastern box turtle and Eastern gray squirrel tracking. The contributions of these works mainly rely on the embedded system. In addition, it does not exist any available biologger — opened or not — dedicated to jellyfish study. The work presented in this publication is in the continuity of [11], [12] and describe all the elements required to manufacture and deploy the first electronic floating tag for tracking and recovery of jellyfish. These elements are the hardware (electronics and mechanics for packaging and attachment) and the resources (hardware and software) for data transmission, storage and display.

## 2 Hardware description

### 2.1 Overview

The tracking platform combines a lightweight floating tag attached to a jellyfish using a tether and a rubber band (Fig. 1) with a shore-based LoRaWAN reception and data-delivery chain (Fig. 2). The tag periodically acquires GNSS positions and transmits them in near real time through LoRaWAN, enabling field monitoring and float recovery during deployments. The LoRaWan transmission relies on The Things Network (TTN), while data gathering and storage are done using a server embedded in a Raspberry Pi. To enable the recovery of the tag and the animal in the field, data are transmitted to a Discord client installed on any kind of connected mobile device.

**Fig. 1:**
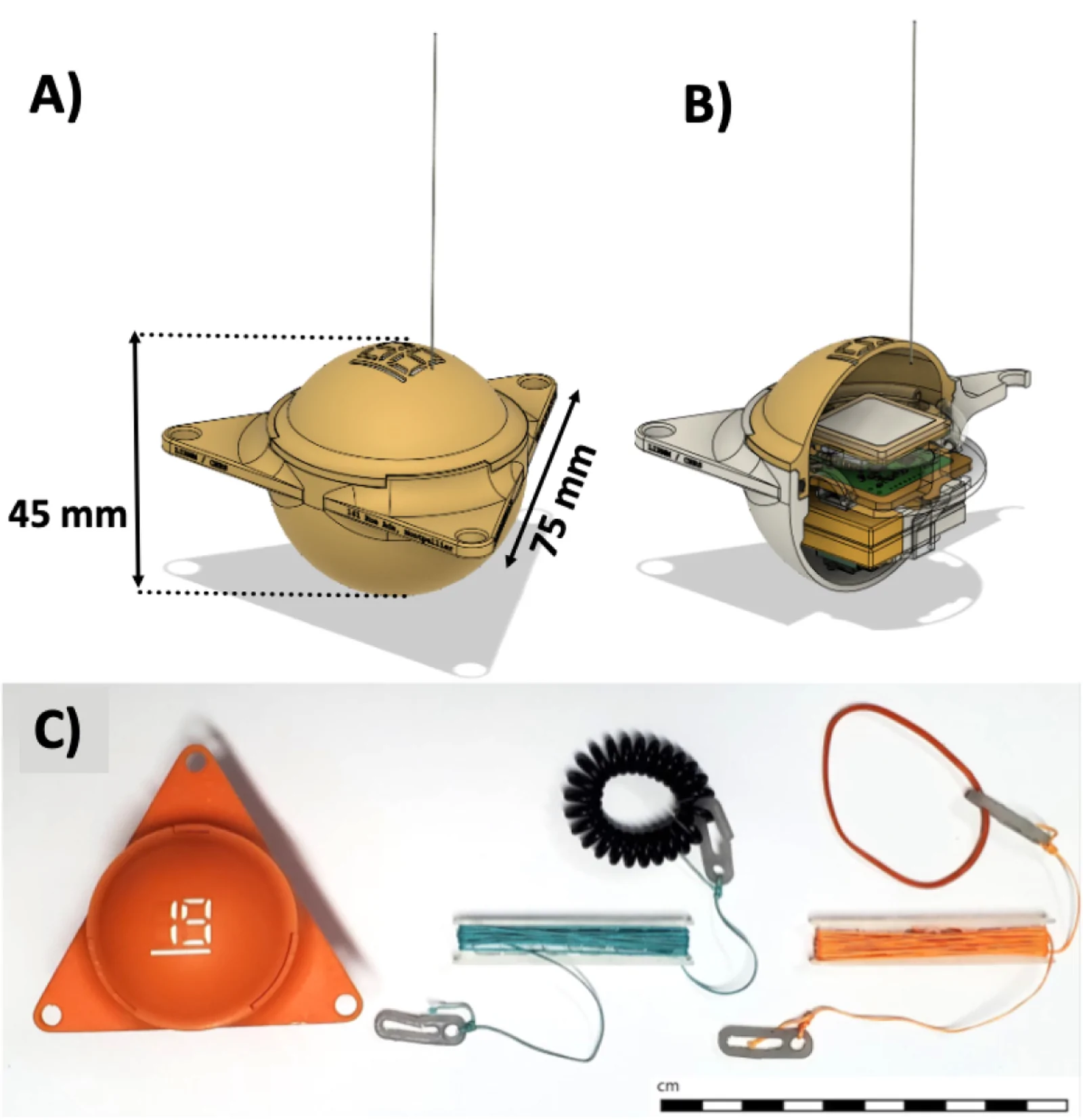
(A) biologger, (B) open view of the biologger, (C) top view of the biologger and two different attachment systems.

**Fig. 2:**
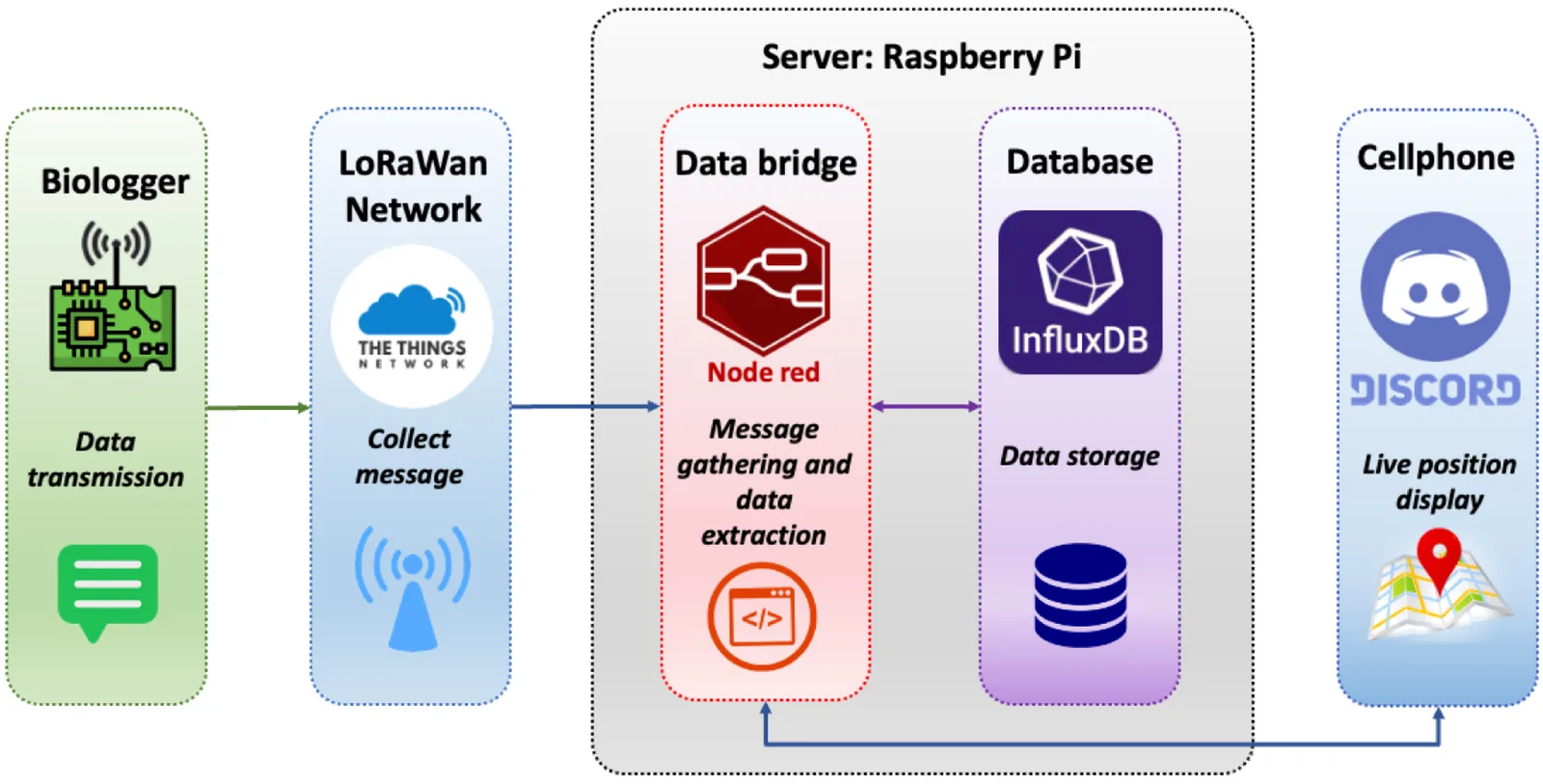
Hardware and software chain.

### 2.2 Floating electronic tag

The floating tag is built around the GNAT LoRa/GNSS asset tracker board (Tlera Corp.) and is integrated into a 3D-printed spherical float (45 mm diameter, 41 g total mass). The GNAT board combines an STM32L082 ultra-low-power microcontroller and an SX1276 LoRa transceiver (CMWX1ZZABZ-078 module), a u-blox MAX-M8Q GNSS receiver, and a LIS2DW12 accelerometer. Although the accelerometer is available on the hardware, it is not exploited in the present study. Indeed the device is a surface float connected to the animal, inertial measurements cannot be directly interpreted as jellyfish body motion without additional instrumentation and calibration. The core sensing and telemetry functionalities used here are GNSS positioning and LoRaWAN transmission.

The system is powered by a rechargeable 3.7 V lithium-ion battery pack made of two separate 155 mAh cells connected in parallel (310 mAh total nominal capacity). The firmware is designed around a low-duty-cycle operation: the tag remains in deep sleep between measurement cycles, then wakes up to perform GNSS acquisition during up to 90 s with an early-stop algorithm based on fix quality. The algorithm is presented in [13]. This algorithm is only used to find a trade-off between power consumption and GNSS position accuracy in a context of battery life optimization. The GNSS position acquisition can also be done using a regular process consisting in repeated fixes over a pre-defined time resulting in getting the last one as the potential best position. Then the tag transmits a compact payload via LoRaWAN, and returns to sleep. This architecture enables multi-day deployments while keeping the device small and buoyant.

Antennas are fully integrated within the enclosure (Fig. 1.(B)). The GNSS ceramic patch antenna (Siretta ECHO19/0.1M/UFL/S/S/ 17) is mounted on an internal support to maintain a stable orientation, while the flexible nitinol-based LoRa antenna is embedded and sealed within the casing using resin to improve robustness in a marine environment and reduce mechanical stress on the RF connexion. The lid-mounted LoRa antenna is not a purchased single part but a simple custom subassembly made from the coaxial cable, nitinol wire, ferrule, and sealing resin.

To simplify charging and handling of the battery pack in the field, all tags include a small custom interface PCB providing an on/off switch and dedicated charging connector compatible with an external single-cell Li-ion charger. This interface avoids repeated manipulation of small battery connectors inside the sealed enclosure and reduces the risk of connector wear or wiring failures during repeated deployment cycles. The PCB design files (schematic/PCB layout) and assembly instructions are provided in the open-source repository.

### 2.3 Data infrastructure and pipeline

Uplink messages are received by a LoRaWAN network using a TTN-compatible gateway, in our case the choice has been made of a Milesight SG50 unit (cf. Fig. 3) for the reason that it is combined with a battery and a solar panel which make it suitable for outdoor deployment even in an off-grid area. Furthermore, this enables a 4G connection when internet network is unavailable. The network server used in this study is The Things Network v3 (EU868), and the tag is provisioned using OTAA. Radio parameters (e.g., spreading factor) are firmware-configurable; in field deployments, the tag was typically operated at SF12 to maximize transmission range.

Uplinks are forwarded from TTN v3 via MQTT to a Node-RED flow running on a Raspberry Pi. This service decodes the compact custom payload (format and decoder provided in the project repository), timestamps and stores received messages in an InfluxDB time-series database, and triggers downstream notifications and visualization. For field operations, the latest positions and trajectory maps are automatically posted to a Discord channel associated with the tagged jellyfish (Fig. 4), enabling straightforward monitoring and recovery using a smartphone.

As a radio-independent fallback, the tag logs the timestamps and GNSS positions to the STM32 internal flash memory during operation. After device recovery, flash logs can be dumped through a dedicated utility firmware and exported to CSV for offline parsing and analysis, allowing reconstruction of the deployment timeline even when uplinks are partially missing.

**Fig. 3:**
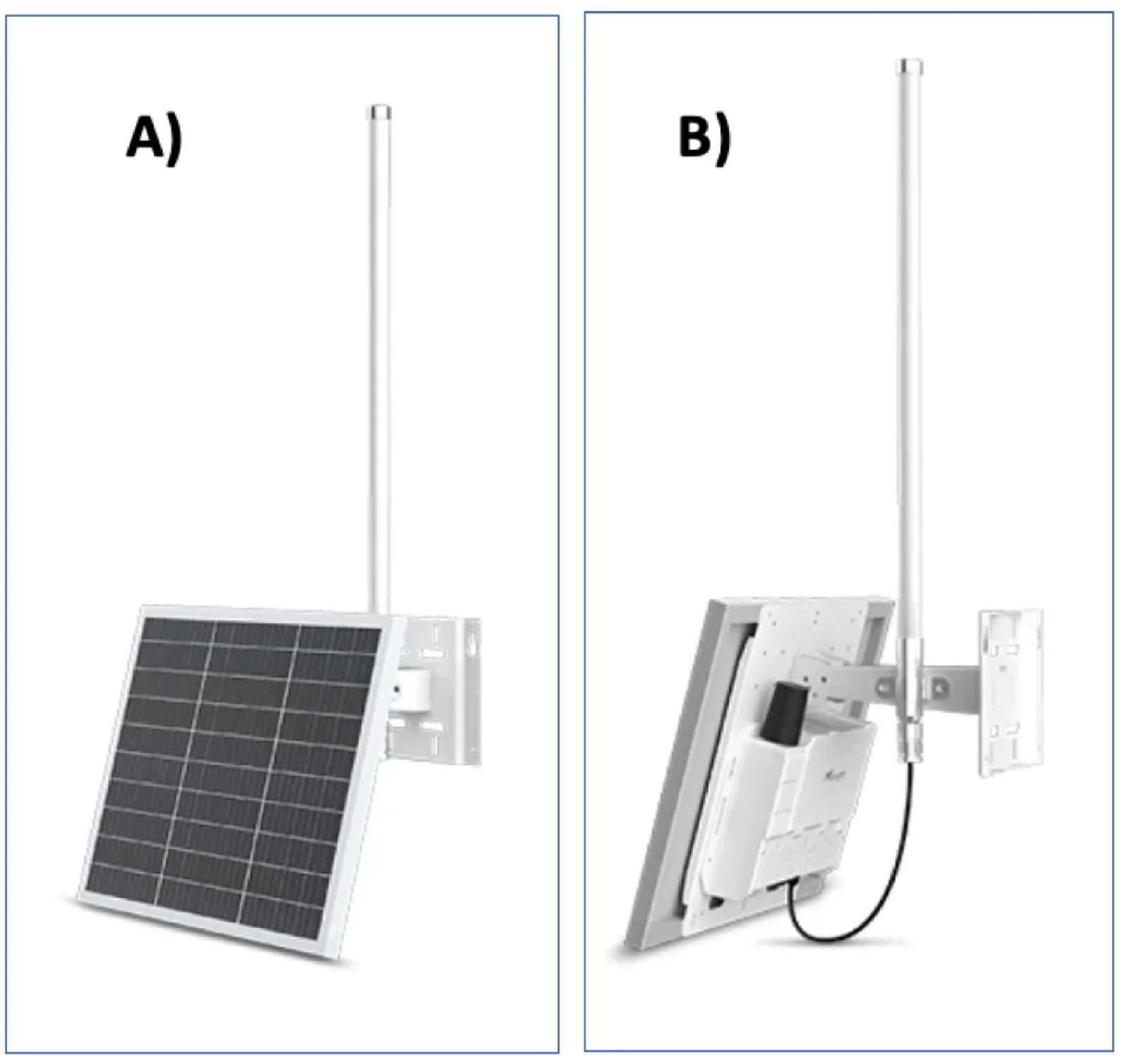
Milesight SG50 Solar powered LoRaWan Gateway, (A) front side of the solar panel and antenna, (B) back side of the solar panel with the gateway and the connection to the antenna.

**Fig. 4:**
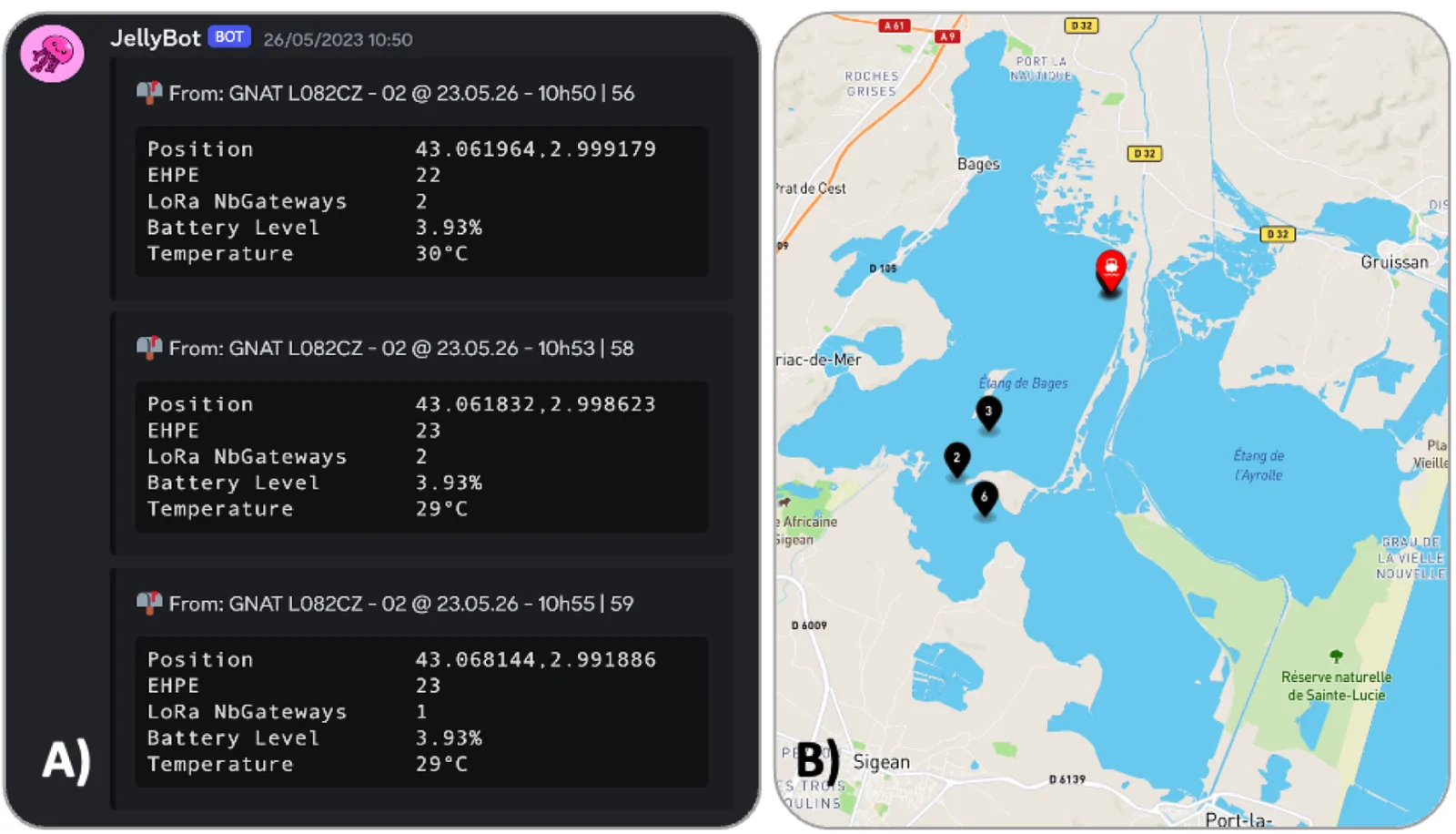
(A) examples messages transmitted by the biologger and displayed on discord client, (B) example of biologgers position map generated for animal recovery, red dot is the smartphone as black dots are the tags deployed.

• The floating tag has been developed for jellyfish tracking but it can also be used for other applications such as tracking water current.
• The open firmware enables to set different deployment scenarios (duration, GNSS sampling frequency ...) and even to use data from the accelerometer which is embedded in the electronic GNAT board but has not used for jellyfish tracking.
• Internal 3D-printed holders have been designed independently of the casing. It makes the electronic part of the tag easy to assemble and re-use in different tracking scenarios for instance of land animals, which would require a more suitable packaging.
• The data infrastructure and pipeline are used in this case to store and display data collected from the tracking device. The same infrastructure can be used for any LoRaWan connected device and particularly in areas with limited network coverage as it relies on a self-powered gateway. For instance, it has been used for gathering data from a weather station.
• The basic principle of using a Discord client to display on a map the GNSS location stored in the distant server alongside the current user location is simple and efficient solution to recover the device. It is particularly useful in any use case for which the device might move. This assumes that the user has a mobile phone or any connected device with built-in GNSS or GPS.

## 3 Design files

Files located in mechanical folder are used for 3D printing of the tag casing. For waterproofing reasons, it is mandatory to print the casing using a resin printer such as the Formlabs Form 3. The folder electronics contains the files needed to order the PCB from a manufacturer such as PCBWay (https://www.pcbway.com) or JLSPCB (https://jlcpcb.com). There is also mechanical CAD files for inner mechanical elements for integration of electronic boards. The inner mechanical elements can be printed using filament 3D printer. There are three different firmwares stored in firmware folder. The Jellytracker code is the one that must be downloaded in the tag before deployment. Flash_read code must be flashed in the tag after deployment to collect stored data. Flash_Erase_memory code is used to erase tag memory after data extraction (see Table 1, Table 2).

**assembly_manual_jellyfish_float_en.pdf:** this file provides the manual step-by-step for the electronic tag assembly.

**Table 1:** Design files summary.

Design filename	File type	Open source license	Location of the file
assembly_manual _jellyfish_float_en	.pdf file	**CC-BY-SA-4.0**	https://doi.org/10.5281/zenodo.21811841
electronics	project folder	**CERN-OHL-S-2.0**	https://doi.org/10.5281/zenodo.21811841
firmware	project folder	**MIT**	https://doi.org/10.5281/zenodo.21811841
mechanical	project folder	**CERN-OHL-S-2.0**	https://doi.org/10.5281/zenodo.21811841
node-red	project folder	**MIT**	https://doi.org/10.5281/zenodo.21811841

**electronics:** this folder contains the KiCad source files used to generate manufacturing files for the battery management board that is used for in-parallel interconnection of two batteries.

**firmware:** this folder contains the three .ino files that can be uploaded in the GNAT board. JellyTracker.ino is the firmware used for the deployment of the tag. Flash_Read.ino is the firmware coded for get data out of the tag memory after a deployment. Flash_EraseMemory.ino is the code to erase memory before a deployment. For each of the three firmwares, the folder contains three sub-folders names src which contain the libraries needed for each firmware.

**mechanical:** this folder contains the STL files, f3d files and step files for each of the five elements that must be 3D printed (board holder, electronic holder, float bottom, float top and GPS holder).

**node-red:** this folder contains the flows.json file. This file is the node-red code used on the raspberry-pi file to collect LoRaWan data, parse and store them in a database.

## 4 Bill of materials

The bill of materials summary below includes the main purchased parts, custom fabricated parts, and assembly consumables required to build one jellyfish tracking float and to collect, store and display using one LoRaWan gateway. For large-area deployments, additional gateways may be required to cover the whole area.

The cost for materials needed to fabricate the tag is 136.73 €. In addition, for charging the device, the charger and the wires cost 5.59 €. For data collection, storage and display, there is a need for a gateway and raspberry Pi 4 for a total amount of 1091 €. The total amount is 1233.32 €.

**Table 2:** Bill of materials summary for one jellyfish tracking float.

Designator	Component	No.	Unit cost	Total cost	Source	Material type
E1	GNAT-L082CZ LoRa/GNSS board (Tlera Corp.)	1	69.55 €	69.55 €	Tindie.com (ref: Gnat LoRa+GNSS Asset Tracker)	Semiconductor

E2	Custom battery-management PCB	1	5 €	5 €	Fabricated from project KiCad files	Semiconductor

E3	LiPo battery, 3.7 V, 155 mAh	2	13.80 €	27.6 €	Farnell	Composite

E4	GNSS antenna	1	14.92 €	14.92 €	Mouser (ref: Siretta ECHO19/0.1M /UFL/S/S/17)	Ceramic

E5	Magnetic micro-USB connector	1	1.99 €	1.99 €	Amazon	Composite

E6	Coaxial cable with male or equivalent μFL connector	1	4.80 €	4.80 €	Mouser (ref: Molex 73412-0237)	Composite

E7	Nitinol wire for antenna	1	27.56 €/spool of 1 m	1.2 €	Amazon (Ref: XEINNILL NiTI wire 0.5 mm)	Metal

E8	Wire ferrule, 20 AWG, 8 mm	1	9.90 €/box	0.02 €	Electrical ferrule supplier/Amazon	Metal

E9	O-ring sealing gasket ID 37.5 mm x 2 mm	1	1.02 €	1.02 €	123roulement.com (Ref: OR-37.50X2-NBR70)	Polymer

E10	Micro USB Li-Po Battery Charger Module, configured for 50 mA (TP4056X, 50/200/500 mA)	1	5.03 €	5.03 €	DFRobot (ref: DFR0667)	Semiconductor

E11	Wire jumper, receptacle to receptacle, 150 mm	2	0.28 €	0.56 €	Farnell (ref: MIKROE-511)	Composite

E12	Server for data gathering and storage	1	130 €	130 €	Amazon.com (Ref: raspberry Pi 4)	Semiconductor

E13	LoRaWan Gateway	1	961 €	961 €	sparwan.com (Ref: Milesight SG50)	Composite

M1	GNAT board holder (3D printed)	1	185 €/L	0.74 €	Printed from repository design files	Polymer

M2	Internal electronics holder (3D printed)	1	159 €/L	1.43 €	Printed from repository design files	Polymer

M3	GNSS antenna holder (3D printed)	1	185 €/L	0.56 €	Printed from repository design files	Polymer

M4	Float bottom shell (3D printed)	1	185 €/L	4.81 €	Printed from repository design files	Polymer

M5	Float top/lid (3D printed)	1	185 €/L	2.59 €	Printed from repository design files	Polymer

C1	Resin or epoxy for antenna sealing	1	159 €/L	0.32 €	Formlabs	Polymer

C2	Cyanoacrylate adhesive	1	1600 €/L	approx 0.16 €	RS Components (Loctite 406, 20 g)	Polymer

C3	Hook-up wire for battery and PCB connections (2 × 60 mm, red and black)	1	0.137 €/m (red), 0.143 €/m (black)	0.02 €	RS Components (UL1007, 28 AWG)	Composite

## 5 Build instructions

The jellyfish tracking float combines custom 3D-printed mechanical parts with a GNAT-L082CZ board (E1), a custom battery-management PCB (E2), two lithium-polymer batteries (E3) connected in parallel, a GNSS antenna (E4), a magnetic USB connector (E5), and a lid-mounted custom LoRa antenna (E6+E7+E8). The design files required to reproduce the custom parts are listed in Section 3 “Design files summary”, while the corresponding purchased components and consumables are listed in the Bill of materials summary, Section 4.

The assembly is based on a compact internal architecture in which the GNAT-L082CZ board, the battery-management PCB, the batteries, and the antenna connections are integrated inside a 3D-printed enclosure. Because the internal fit is tight, printing quality, cable routing, and assembly order are critical to ensure proper closure and reliable sealing. Some elements, especially the lid-mounted LoRa antenna, can be prepared in advance as subassemblies. A more detailed illustrated assembly manual can be provided in the project repository, but the essential construction steps are given below.

### 5.1 Overview of printed parts and components

The main parts required for assembly are shown in Fig. 5. The printed parts are the float bottom shell (M4), the float lid (M5), the GNAT board holder (M1), the internal electronics holder (M2), and the GNSS antenna holder ((M3). The main purchased components are the GNAT-L082CZ board (E1), the battery-management PCB (E2), the two batteries (E3), the GNSS antenna (E4), the magnetic USB connector (E5), and the O-ring (E9). The lid-mounted LoRa antenna subassembly is fabricated separately from a short coaxial cable (E6), a nitinol wire (E7), and a ferrule (E8), as described in the next subsection.

All printed parts (M3,M4) were produced on a Formlabs Form 3 printer using a layer height of 25 μ m. All parts were printed in Formlabs Color Resin V1 (orange), except the internal electronics holder (M1, M2, M3), which was printed in Formlabs Clear Resin V4. The use of a transparent material for this internal part is not strictly mandatory, but it facilitates visual inspection of the internal arrangement and cable routing during assembly. The selected print resolution was found necessary to obtain sufficiently accurate fits for the compact internal integration and clip-based closure system.

**Fig. 5:**
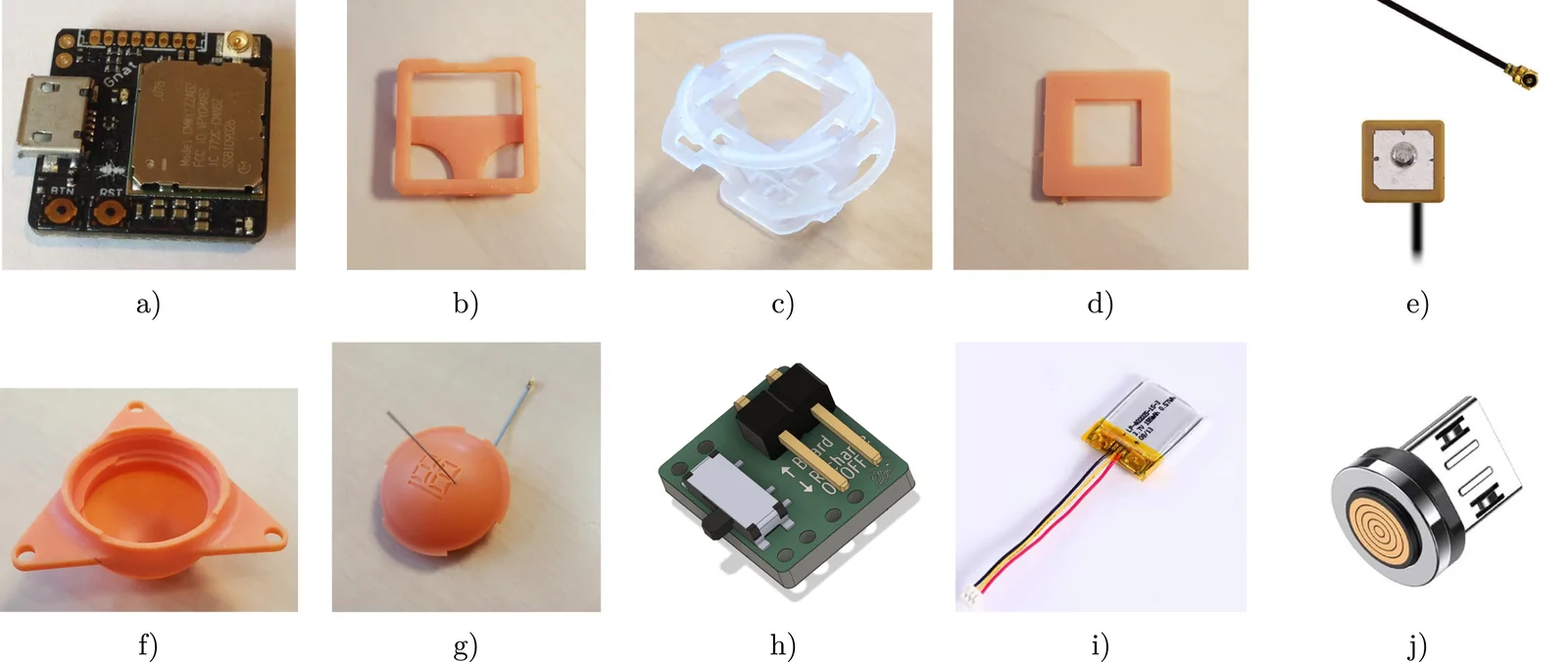
Overview of the main printed parts and components required to assemble the jellyfish tracking float: (a) GNAT-L082CZ board (E1); (b) GNAT board holder (M1); (c) internal electronics holder (M2); (d) GNSS antenna holder (M3); (e) GNSS antenna (E4); (f) float bottom shell (M4); (g) float lid (M5); (h) battery-management PCB (E2); (i) LiPo battery (E3); and (j) magnetic USB connector(E5).

### 5.2 Preparation of the printed parts

After printing, all parts should be cleaned and post-processed according to the resin manufacturer’s recommendations. Any support residues, burrs, or local defects that could interfere with part fitting, cable routing, or sealing should be removed carefully. Particular attention should be paid to the clip interfaces, the O-ring seating surfaces, and the passages used by the GNSS and LoRa cables.

At this stage, users should verify that the GNAT-L082CZ board (E1) fits correctly in the GNAT board holder (M1), that the internal electronics holder can receive the internal components without excessive force, and that the float lid (M5) and float bottom shell (M4) close correctly when empty. Because the final assembly tolerances are tight, dimensional deviations or insufficient post-processing may prevent correct clipping or may create unwanted stress on the internal wiring.

### 5.3 Pre-assembly of the lid-mounted LoRa antenna

The float lid includes a simple custom LoRa antenna made from a nitinol wire mechanically connected to a short coaxial cable (Fig. 6). This subassembly can be prepared in advance before final tag assembly. In the current design, the antenna uses 65 mm of 0.5 mm-section nitinol wire (E7) and a 45 mm coaxial cable terminated with a male μFL connector (E6). A wire ferrule (20 AWG, 8 mm length, E8) is used as a crimped junction between the stripped coaxial core and the nitinol wire. The lengths of coaxial cable and nitinol wire are important because they define the antenna tuning.

To fabricate this subassembly, the plastic sleeve of the ferrule is first removed so that only the metal tube is retained. The end of the coaxial cable is then stripped by removing approximately 10–15 mm of external insulation and shielding, followed by about 5 mm of core insulation. The nitinol wire is inserted fully into the ferrule, while the stripped coaxial core is inserted with approximately 1–2 mm of remaining insulation left near the crimp to provide mechanical protection. The ferrule is then crimped to mechanically join the nitinol wire and the coaxial core.

A small hole is drilled in the float lid at the designed antenna location. The assembled antenna is inserted from the inside of the float lid until the ferrule comes into contact with the lid surface, after which the assembly is sealed in place using Formlabs Clear Resin V4 or a similar resin. Epoxy resin can also be used as an alternative sealing material. Once cured, this subassembly forms the finished float lid used in the subsequent integration steps.

This design was chosen because it provides a simple, lightweight, and compact antenna integration compatible with the float lid geometry. Nitinol is particularly suitable in a marine environment because it is corrosion-resistant and is a shape-memory alloy. The main critical points are the integrity of the coaxial core during stripping and crimping, the accuracy of the drilled hole position, and the quality of the final sealing. Users should take the usual precautions associated with cutting tools, drilling operations, UV-curable or epoxy resins, and sharp metal wire ends.

**Fig. 6:**
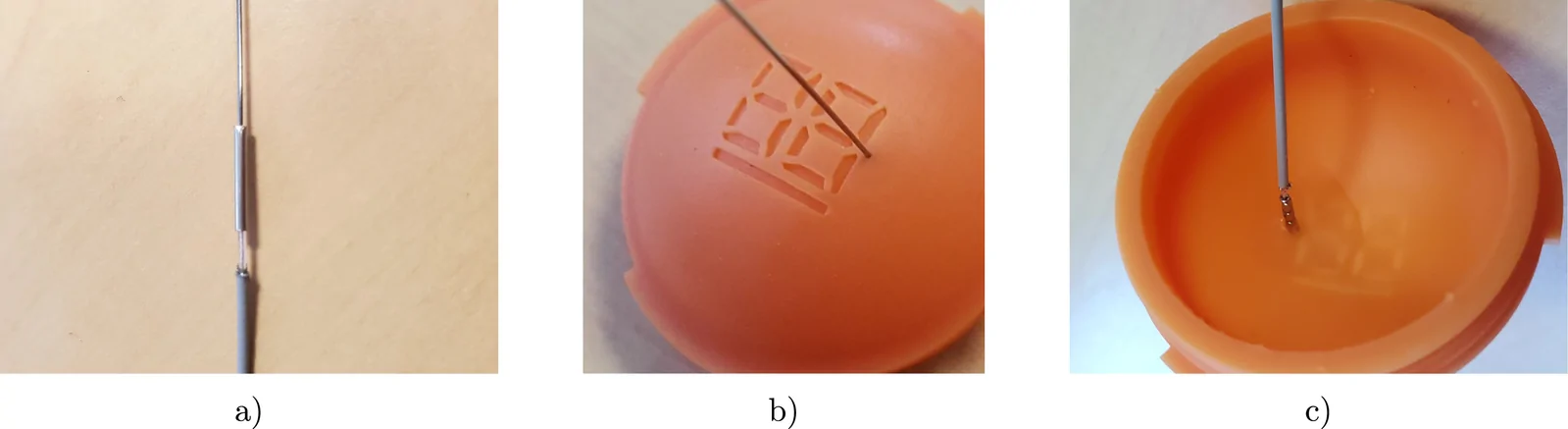
Pre-assembly of the lid-mounted LoRa antenna. The subassembly uses a nitinol wire, a short coaxial cable with male μFL connector, a crimped ferrule junction, and resin sealing inside the float lid; (a) Prepare the ferrule, strip the coaxial cable, and crimp the nitinol wire to the coaxial core; (b) Drill the float lid and insert the antenna from the inside until the ferrule contacts the lid; (c) Seal the antenna in place with resin and obtain the finished float lid subassembly.

### 5.4 Installation of the GNSS antenna

The first assembly stage of the main enclosure is the preparation of the GNSS antenna subassembly (Fig. 7). The GNSS antenna (E4) is first placed onto the GNSS antenna holder (M3). The antenna cable is then routed through the internal electronics holder, after which the GNSS antenna holder is glued onto the internal electronics holder using a standard cyanoacrylate adhesive (C2).

This order is important because the GNSS antenna becomes less accessible once the internal electronics are installed. The glued holder also fixes the antenna position and reduces cable movement inside the enclosure. Care should be taken not to contaminate the antenna surface or the cable with excess adhesive.

**Fig. 7:**
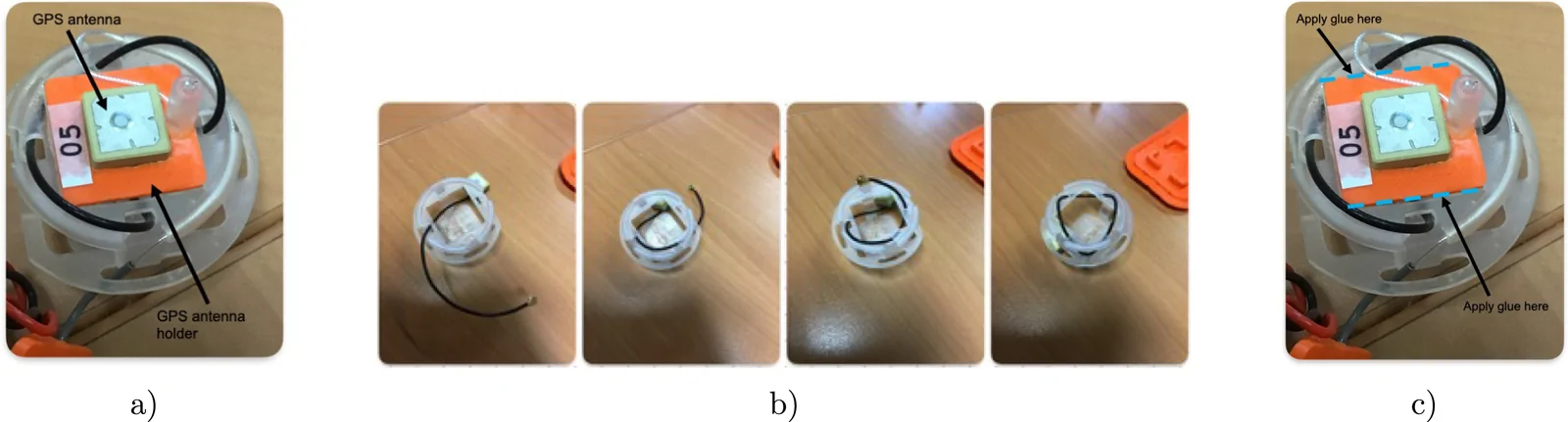
Installation of the GNSS antenna and cable routing prior to insertion of the internal electronics; (a) Place the GNSS antenna on the GNSS antenna holder; (b) Route the antenna cable through the internal electronics holder; (c) Glue the GNSS antenna holder in place.

### 5.5 Assembly of the power subsystem

The battery-management PCB (E2) provides battery charging, power switching, and the electrical interface between the GNAT-L082CZ board and the battery pack. Two LP-402025-1S-3 lithium-polymer batteries (3.7 V, 155 mAh each, E3) are connected in parallel. In the present design, the cable between the GNAT-L082CZ board and the battery-management PCB is 60 mm long.

The two batteries may be taped together to simplify handling before insertion. Their terminals can be cut and soldered directly to the battery-management PCB if needed, which reduces the difficulty associated with soldering flexible stranded wires in a confined assembly. The battery polarity is indicated directly on the PCB silkscreen to facilitate correct wiring during assembly.

Because lithium-polymer cells are used, polarity must still be checked carefully before connection, and soldering should be performed with the usual precautions required for battery-powered circuits. Be careful to avoid mechanical damage, overheating, or short-circuiting the cells during assembly (see Fig. 8).

**Fig. 8:**
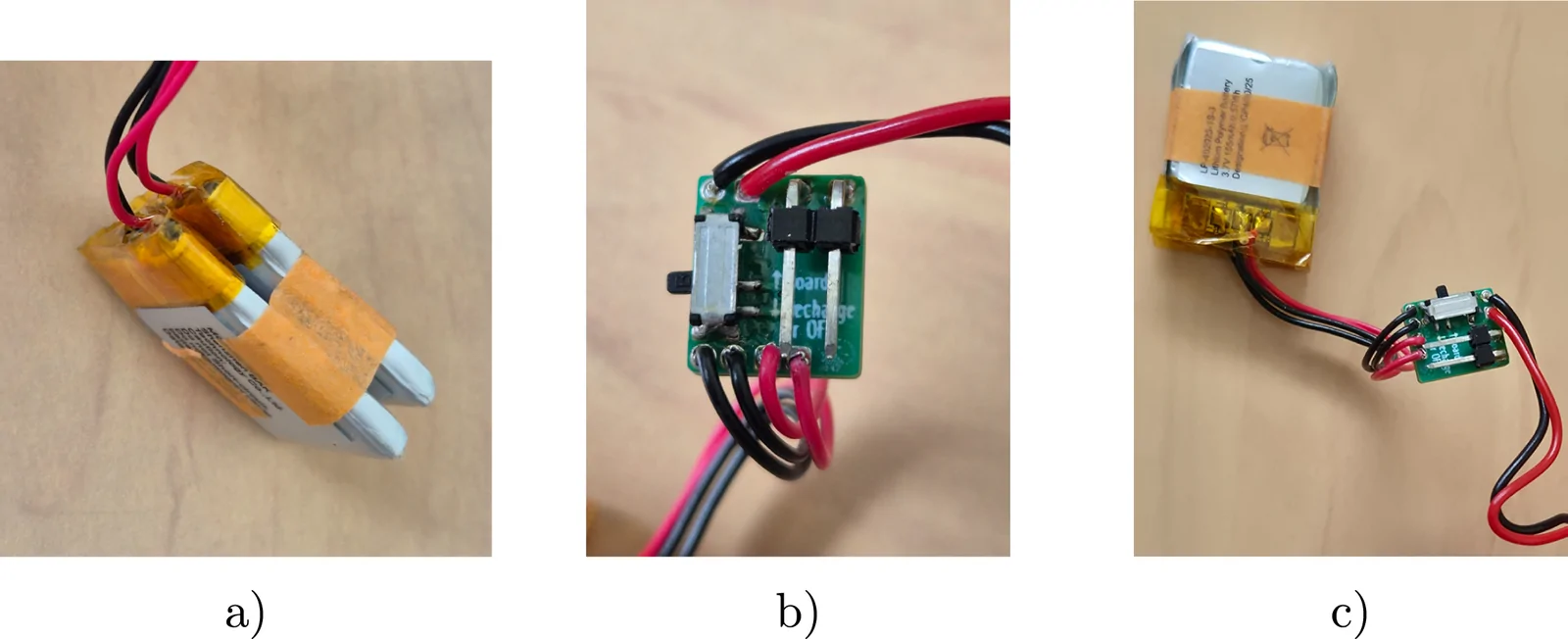
Assembly of the two batteries and connection to the battery management board; (a) Tape the two batteries together; (b) Connect the two batteries to the battery management PCB; (c) Connect the GNAT 60 mm cables, be careful the switch is RECHARGE OR OFF to prevent short circuits.

### 5.6 Integration of the GNAT-L082CZ board and internal electronics

The GNAT-L082CZ board is inserted into the GNAT board holder, after which the battery-management PCB is connected to the GNAT-L082CZ board and to the battery pack (Fig. 9). The GNSS antenna cable is then connected to the GNAT-L082CZ board. After this, the connector coming from the lid-mounted LoRa antenna is attached, and the GNAT-L082CZ board together with the GNAT board holder is clipped into the internal electronics holder. Finally, the battery pack is inserted into the internal electronics holder, with the Kapton side facing downward.

This sequence is preferred because it minimizes cable stress and preserves access to the small connectors before the assembly is fully compacted. The transparent internal electronics holder is especially useful at this stage, as it allows visual inspection of the relative positions of the battery pack, the battery-management PCB, the GNAT board holder, and the internal wiring.

The main design constraint during this step is cable routing. Wires and coaxial connections must remain clear of the clip interfaces and of the enclosure mating surfaces. Poor routing may prevent proper closure, damage connectors, or compromise long-term sealing reliability.

**Fig. 9:**
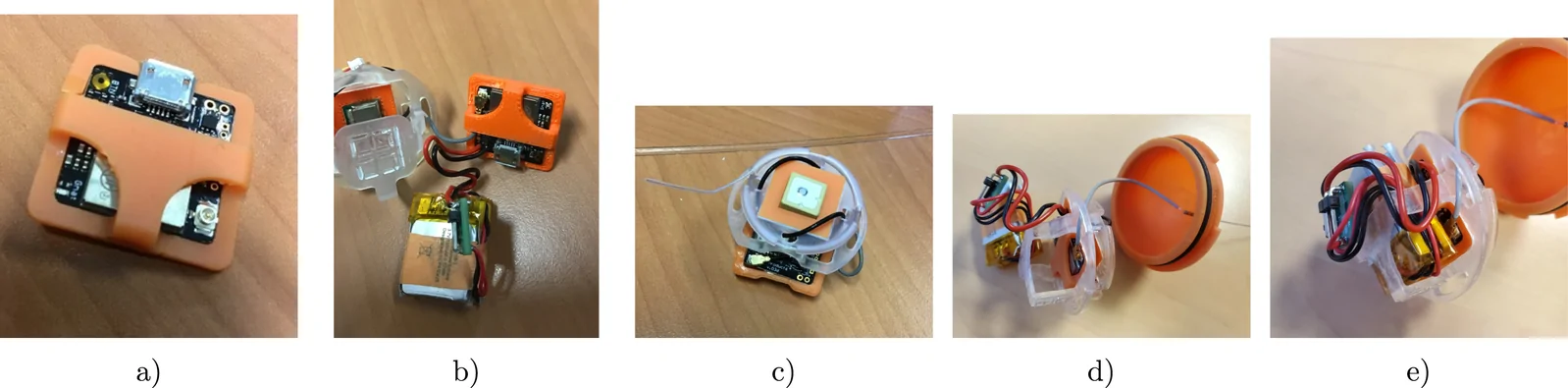
Integration of the internal electronics: insertion of the GNAT-L082CZ board, connection of the power subsystem and GNSS antenna, clipping of the internal assembly, and insertion of the battery pack; (a) insert the GNAT-L082CZ board into the GNAT board holder; (b) connect the battery-management PCB and the two batteries; (c) connect the GNSS antenna to the GNAT-L082CZ board; (d) connect the lid-mounted LoRa antenna and clip the assembly into the internal electronics holder; (e) insert the battery pack with the Kapton side facing downward.

### 5.7 Final enclosure assembly and sealing

Once the internal assembly is complete, the magnetic USB connector (E5) is inserted into the micro-USB port of the GNAT-L082CZ board. The assembled internal unit is then placed inside the float bottom shell, the O-ring is positioned around the float lid, and the enclosure is closed by clipping the float lid into place (Fig. 10).

The O-ring is the main sealing element of the enclosure and should be inspected before closure to ensure that it is clean, undamaged, and properly seated. During final closure, no wire should be pinched between the float lid and the float bottom shell. The clip-based closure was chosen to simplify repeated opening and closing during prototyping and maintenance, while preserving a compact geometry. In the current version, watertightness was evaluated by a static immersion test at 1.5 m depth for 72 h.

**Fig. 10:**
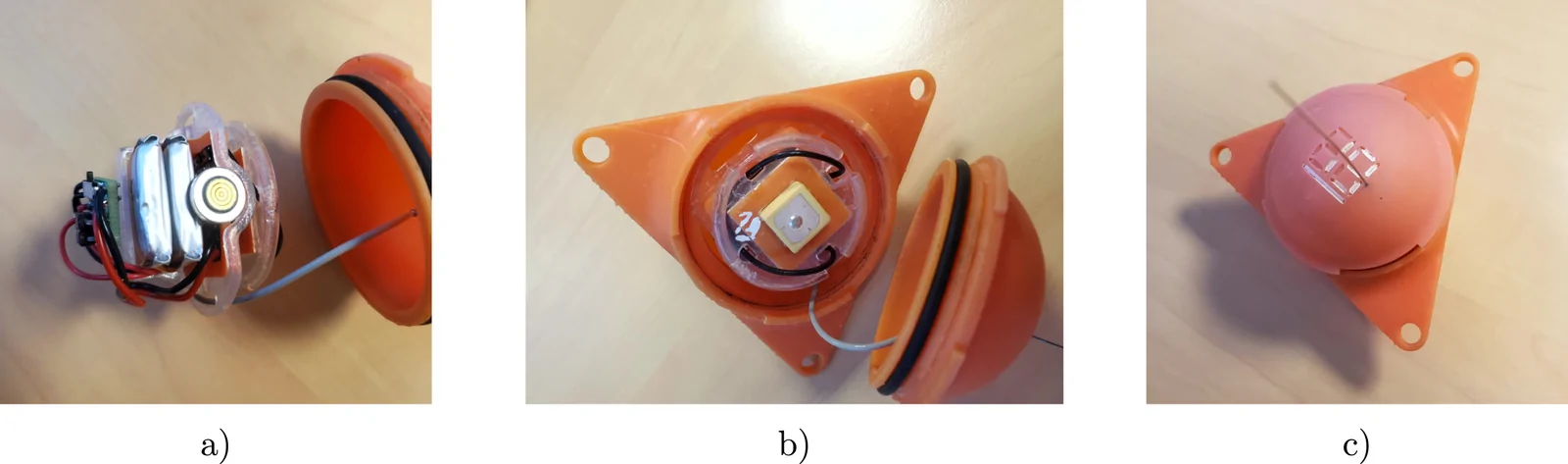
Final enclosure assembly and sealing of the jellyfish tracking float; (a) insert the magnetic USB connector; (b) place the internal assembly into the float bottom shell; (c) add the O-ring and close the enclosure by clipping the float lid into place.

### 5.8 Firmware flashing and pre-deployment checks

The assembled tag is programmed via USB. Before field use, the flash memory region used for onboard data logging should be erased using the Flash_EraseMemory utility firmware. The main JellyTracker firmware can then be uploaded for deployment. After recovery, the Flash_Read utility firmware is used to read out the logged data through the serial interface for external parsing.

Before closing the enclosure for an actual deployment, a basic functional validation should be performed. At minimum, this check should confirm correct power-up, battery charging behavior, firmware upload, GNSS detection, and LoRaWAN transmission. This can be done using JellyTracker firmware with serial monitoring activated.

The main firmware uses periodic RTC-triggered acquisition and low-power operation. The acquisition interval is configurable in the source code. More details are provided in the operation instructions.

## 6 Operation instructions

Once assembled, the jellyfish tracking float is prepared for deployment by charging the batteries, uploading the appropriate firmware sketch, configuring the LoRaWAN credentials, and verifying that the enclosure is properly sealed. During deployment, the device periodically acquires GNSS positions and transmits compact data packets through The Things Network (TTN). If radio transmission is temporarily unavailable, the onboard logging memory can be used as a backup for post-recovery data extraction.

### 6.1 Pre-deployment checks

Before each deployment, the following points should be checked:

• the batteries are fully charged;
• the GNSS antenna and the lid-mounted LoRa antenna are correctly connected;
• the correct firmware sketch has been uploaded to the GNAT board;
• the DevEUI, AppEUI, and AppKey stored in the firmware match the TTN device configuration;
• the enclosure is dry and free of visible damage;
• the O-ring is clean, correctly positioned, and not damaged;
• the lid closes fully and the enclosure is properly sealed.

A short functional test should be performed before field deployment to confirm that the device powers correctly, joins TTN, and produces a first uplink.

### 6.2 Battery charging and device preparation

The float is powered by two lithium-ion cells connected in parallel through the battery board. Before charging, inspect the device for visible damage, water ingress, or battery swelling. Charging requires the toggle switch to be put in the POWER OFF/CHARGE position and should only be performed in dry conditions using an appropriate lithium-ion charger.

After charging, the magnetic USB connector can be used for firmware upload and configuration. Once the device is ready for deployment, the toggle switch must be put in the POWER ON position, and the enclosure should be closed with the O-ring in place.

The following points should be checked carefully before closure:

• the O-ring is not twisted;
• no cable interferes with lid closure;
• the GNSS and LoRa connectors remain fully seated during handling;
• no moisture is present inside the enclosure.

### 6.3 Firmware installation and sketch selection

The GNAT-L082CZ board is programmed using the Arduino IDE together with the STM32L0 Arduino core by GrumpyOldPizza (https://github.com/GrumpyOldPizza/ArduinoCore-stm32l0). The core explicitly supports the Gnat-L082CZ board and can be installed through the Arduino Board Manager by adding the STM32L0 package index URL in the Arduino IDE preferences, installing the Tlera Corp STM32L0 Boards package, and then selecting the Gnat-L082CZ board in the Tools -> Board menu.

The repository contains three firmware sketches:

• JellyTracker/, the main mission firmware used during normal deployments;
• Flash_Read/, a utility sketch used after recovery to read the stored data from onboard memory;
• Flash_EraseMemory/, a utility sketch used to erase previously stored data before a new deployment cycle.

The normal firmware workflow is the following:

1. upload JellyTracker before field deployment;
2. use Flash_Read only after float recovery, when stored data need to be extracted;
3. use Flash_EraseMemory only after confirming that the stored records are no longer needed;
4. re-upload JellyTracker before any new field deployment.

Uploading either Flash_Read or Flash_EraseMemory temporarily replaces the main mission firmware. The device must therefore be flashed again with JellyTracker before reuse in the field.

### 6.4 Configuration of the main mission firmware

Before compiling and uploading JellyTracker, the user must replace the publication-safe dummy LoRaWAN identifiers with valid credentials for the intended TTN device. In the present implementation, the parameters that must be checked before compilation are:

• DevEUI;
• AppEUI;
• AppKey;
• the RTC alarm interval used to schedule periodic acquisitions;
• the selected GNAT index (GNAT_L082CZ_) if a table of DevEUI values is used.

The acquisition interval is defined in the RTC alarm configuration. In the current code base, predefined alarm periods include 2 s, 10 s, 30 s, 90 s, 2 min, 5 min, 10 min, 15 min, 20 min, 25 min, and 30 min. The nominal setting used in the present system is 10 min.

The GNSS configuration does not normally need to be modified for standard use. In the present implementation, the receiver is configured at 1 Hz with the GPS, GLONASS, and GALILEO constellations enabled, using the SEA platform profile and an external antenna. The GNSS acquisition strategy is split into two phases. At start-up, a dedicated first-fix routine is executed before the beginning of normal logging. This routine repeatedly calls short GNSS acquisition windows in order to allow the receiver to stabilize before deployment monitoring begins. In the current firmware version, this initial phase lasts approximately five minutes.

During normal operation, each acquisition cycle uses a bounded GNSS search window. In the current implementation, the nominal maximum GNSS on-time is 90 s. However, the final position selection does not rely only on a simple EHPE threshold. Instead, the firmware evaluates successive fixes and applies an early-stop strategy based on fix quality and stability: a high-quality fix is accepted immediately, an intermediate-quality fix is accepted after repeated spatially stable solutions, and lower-quality fixes are retained only if no better solution is obtained. A maximum number of valid fixes per cycle is also enforced. The GNSS power-up procedure also includes a recovery mechanism. If the receiver remains busy for too long during activation, the firmware power-cycles and reconfigures the GNSS module before continuing.

Regional radio settings are defined in src/LoRaWAN/LoRaWAN.cpp, in the Config_And_JoinOTAA(...) function, through a call of the form LoRaWAN.begin(REGION). In the current firmware version, the region is set to EU868. Changing the radio region therefore requires editing this function and replacing begin(EU868) with the desired regional setting supported by the library. The same function also defines additional LoRaWAN parameters such as adaptive data rate, data rate, transmission power, and sub-band selection. Users modifying the radio region should verify that these associated settings remain consistent with the selected regional plan.

### 6.5 Flashing procedure

The standard flashing procedure is:

1. Connect the GNAT-L082CZ board to the computer through the USB interface.
2. Open the Arduino IDE.
3. Confirm that the STM32L0 core and the Gnat-L082CZ board are correctly selected.
4. Open the sketch corresponding to the intended task:
  • JellyTracker for deployment,
  • Flash_Read for data extraction,
  • Flash_EraseMemory for memory clearing.
5. If JellyTracker is used, update the TTN credentials and, if necessary, the RTC alarm interval or GNAT board index.
6. Compile the sketch.
7. Upload the sketch to the board.
8. Verify correct behavior after flashing.

After uploading JellyTracker, correct operation should be confirmed by checking that:

• the device successfully joins TTN;
• a first uplink is received after the selected logging period.

If a faulty sketch prevents normal USB-based programming, the STM32L0 core documentation recommends forcing the board into bootloader mode by toggling RESET while holding BOOT, and then reflashing a known working sketch.

### 6.6 Recovery sketches and onboard logging memory

If radio reception was incomplete during deployment, the Flash_Read sketch can be uploaded after float recovery. This sketch outputs the stored records as semicolon-separated serial text suitable for later parsing.

Each acquisition is stored as a fixed-length 20-byte record comprising five 32-bit words. The stored data include the GNSS-derived date and time (YYMMDDHHMM, with seconds stored separately), latitude and longitude encoded as integers with a resolution of 10^−6^ degree, the number of satellites, estimated horizontal and vertical position errors (EHPE and EVPE), GNSS fix type and fix quality, battery voltage, and the LoRaWAN busy and joined status. In the firmware version used for the field deployments, a fixed UTC+2 offset is applied to the GNSS time before storage.

The flash region reserved for onboard logging provides 59,008 bytes of storage. With a record size of 20 bytes, its theoretical capacity is 2950 acquisition records. With the current two-battery configuration, battery endurance is reached before this storage capacity is exhausted. Endurance tests resulted in measured operating durations of approximately 1.5 days at a 2-min acquisition and transmission interval, 4 days at a 10-min interval, and 6 days at a 15-min interval. Consequently, practical deployment duration is currently limited primarily by battery capacity rather than onboard storage capacity.

In the current workflow, the serial output includes a header line. Because some metadata, such as the GNAT identifier or the DevEUI, are not stored directly in the logged records, this header may need to be updated manually before parsing. In addition, the header definition should be kept consistent with the actual logged columns used in the current firmware version.

Once the stored data have been safely recovered, the Flash_EraseMemory sketch can be uploaded to clear the memory reserved for onboard data logging. In the present implementation, this operation erases a dedicated region of the internal microcontroller flash used for data storage, then rewrites the initial pointer value used to mark the beginning of the logging area. The operator should therefore only run this sketch after confirming that all required records have been retrieved and archived.

Because data logging relies on a reserved internal flash region, excessive firmware growth may reduce the memory margin available for storage. Large code modifications should therefore be avoided unless memory usage is re-evaluated.

### 6.7 Field deployment

Immediately before deployment, the operator should verify that the enclosure is properly sealed and that the device has completed its initial start-up sequence.

The float can then be attached and deployed according to the biological protocol used in the study. After release, the first uplinks should be checked to confirm that the device is operating normally.

During the mission, the received position messages can be monitored through the communication and processing chain. In the present implementation, this chain includes:

• TTN as the LoRaWAN network server;
• MQTT forwarding;
• decoding and routing in Node-RED;
• storage in a time-series database.

This workflow allows real-time field supervision and facilitates float recovery.

### 6.8 Post-recovery procedure

After recovery, the device should be handled according to the following sequence:

1. place the toggle switch in the POWER OFF/CHARGE position immediately after recovery to stop acquisition;
2. rinse the device with fresh water if it was used in seawater;
3. dry the exterior before further handling;
4. inspect the enclosure for leakage, mechanical damage, connector degradation, and O-ring wear;
5. if necessary, upload Flash_Read and save the serial output;
6. once the data have been secured, upload Flash_EraseMemory if memory clearing is required;
7. re-upload JellyTracker before the next field mission.

### 6.9 Common failure modes and safety considerations

The main safety considerations associated with the device operation concern the lithium-ion battery, the waterproof integrity of the enclosure, and the potential irreversible loss of logged data. To ensure safe operation, damaged or swollen batteries must not be used, and battery charging must only be performed under dry environmental conditions. In addition, the enclosure must not be sealed when moisture is detected inside the float, as water ingress may compromise both the electronics and the battery safety. The firmware command Flash_EraseMemory, which permanently deletes all stored onboard records, should only be executed after confirming that all relevant data have been successfully recovered.

The most frequently encountered causes of malfunction are related to firmware configuration, communication interfaces, antenna integration, and mechanical assembly. In particular, incorrect The Things Network (TTN) credentials embedded in the firmware or inconsistencies between the firmware configuration and the TTN device settings may prevent communication. Additional failure modes include incomplete antenna connections, poor GNSS antenna coupling, improper positioning of the O-ring seal, incomplete closure of the enclosure lid, and accidental deployment of a maintenance firmware sketch instead of the validated deployment firmware.

## 7 Validation and characterization

The hardware was validated as an open-source field platform for jellyfish tracking in shallow coastal environments. Validation addressed both the floating tag itself and the associated data chain, from onboard GNSS acquisition to LoRaWAN transmission and downstream field monitoring tools.

### 7.1 Scientific application and field use case

The main scientific application targeted by the system is the tracking of jellyfish displacement in coastal lagoons using a lightweight floating relay attached to the animal.

Three TTN-compatible gateways (Milesight SG50) have been deployed around the Bages-Sigean lagoon, France. During field deployments, gateway antennas were installed at approximately 5–10 m above ground to improve line-of-sight over the water and provide coverage over the study area. The choice for the location of gateways, presented in Fig. 11, is a trade-off between the maximum coverage for each antenna and the legal agreement obtained from the responsible authorities (municipalities and coastal protection agency).

The Bages-Sigean lagoon has been chosen because since 2014, it is the site of annual summer blooms of *Rhizostoma pulmo* which makes it suitable for jellyfish tagging operations. The lagoon is 38 km^2^, with an average of 2-meter depth.

**Fig. 11:**
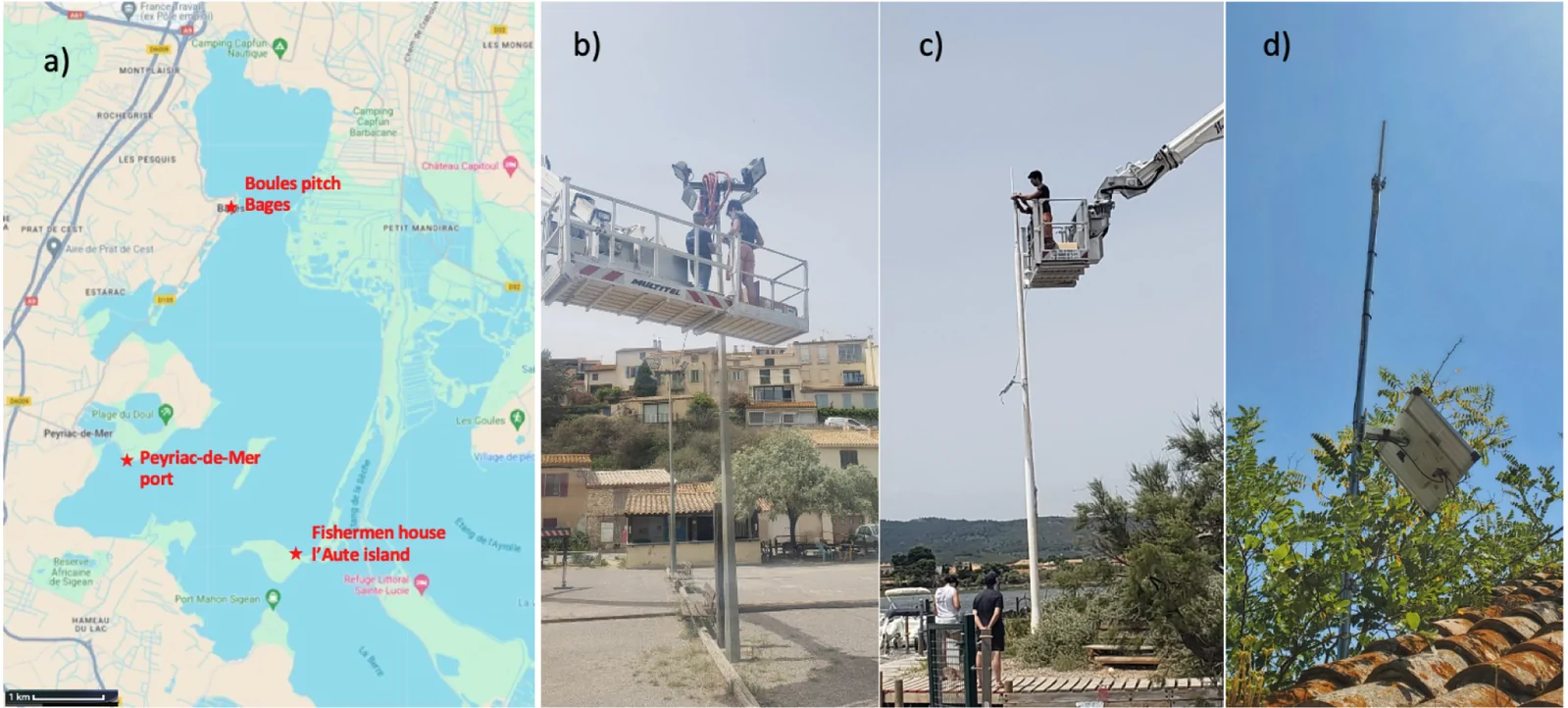
(a) map of the Bages-Sigean lagoon with the location of the three gateways, (b) installation of gateway on boules pitch at Bages, (c) installation of gateway at Peyriac-de-mer port, (c) gateway on roof of fishermen house at l’Aute island.

Jellyfish collection was conducted with a fisherman boat. As presented in Fig. 12, R. pulmo medusae were first visually located and then captured with a handnet. Afterwards, the tagging device was attached to the manubrium by carefully passing the oral arms through the stretched elastic band. The fishing line connects the floating tag to the elastic attachment system. The fishing line used as a tether between the tag and the jellyfish is 3 m long, which enables the animals to dive as the lagoon depth averages 2 m deep. To avoid any potential buoyancy modification induced by air bubbles injected under the bell, jellyfish were kept submerged during the tagging operation.

**Fig. 12:**
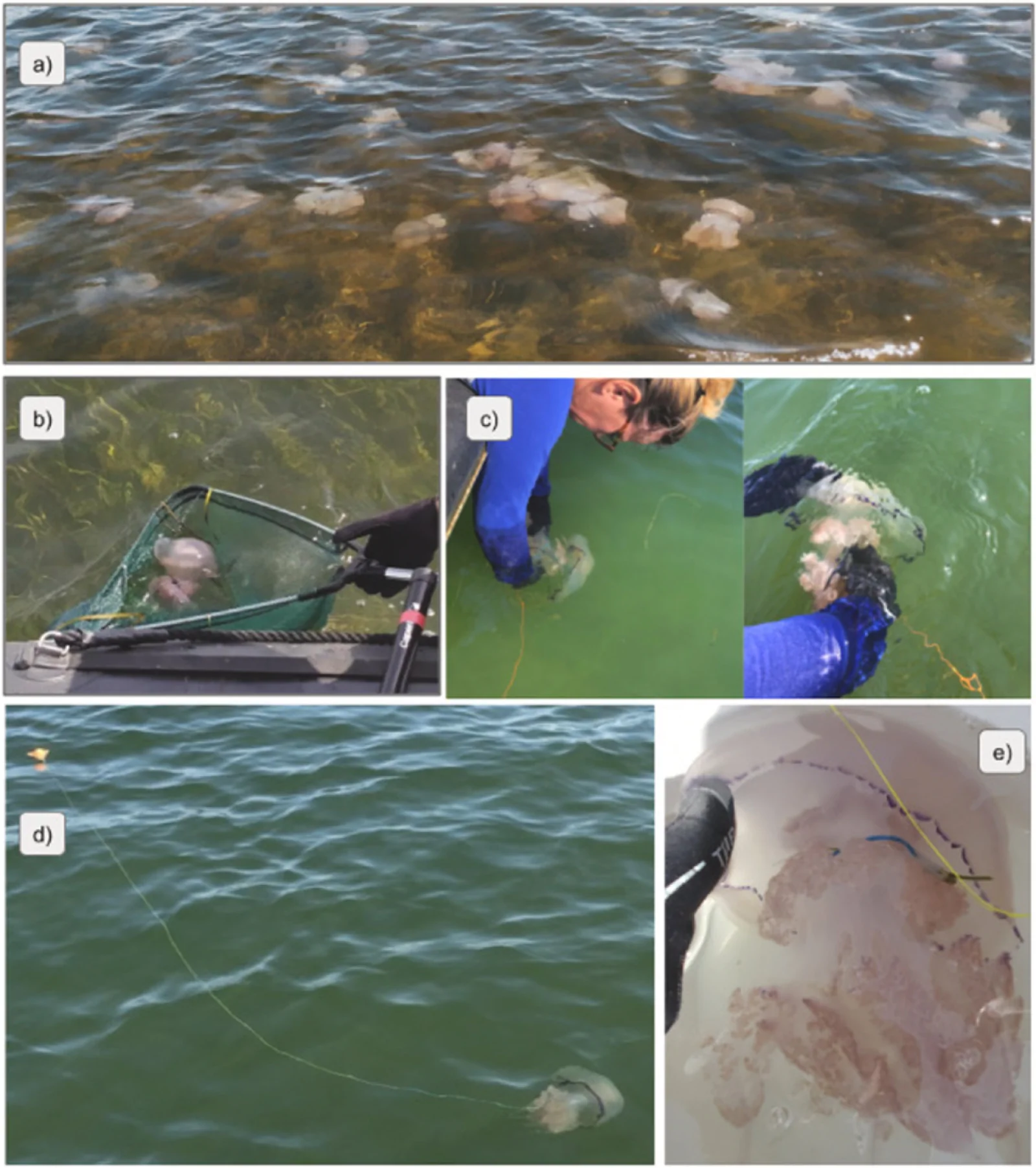
In situ tagging technique : (a) Jellyfish spotting, (b) Jellyfish carefully collected using a handnet, (c) Jellyfish handled in the water for biometry and tag attachment, (d) release of the tagged jellyfish, (e) recovered tagged jellyfish.

### 7.2 End-to-end system validation

A maximum number of 12 tags and 3-gateway network were used over two deployment seasons, from May to October 2024 and 2025. This resulted in 13 jellyfish tagged in 2024 and 30 jellyfish tagged in 2025.

Observed deployment duration ranged from about 1 h to 96 h. This range covers both short engineering tests and multi-day biologging deployments.

The complete tracking chain was validated in real field conditions. During operation, the device woke from low-power sleep, acquired a GNSS position onboard, encoded a compact telemetry packet, and transmitted it through a LoRaWAN uplink. Messages were received through The Things Network (TTN), then exported through MQTT to downstream monitoring tools. In the field implementation used here, these tools supported data storage and operator notifications, including Discord notifications during field operations.

**Fig. 13:**
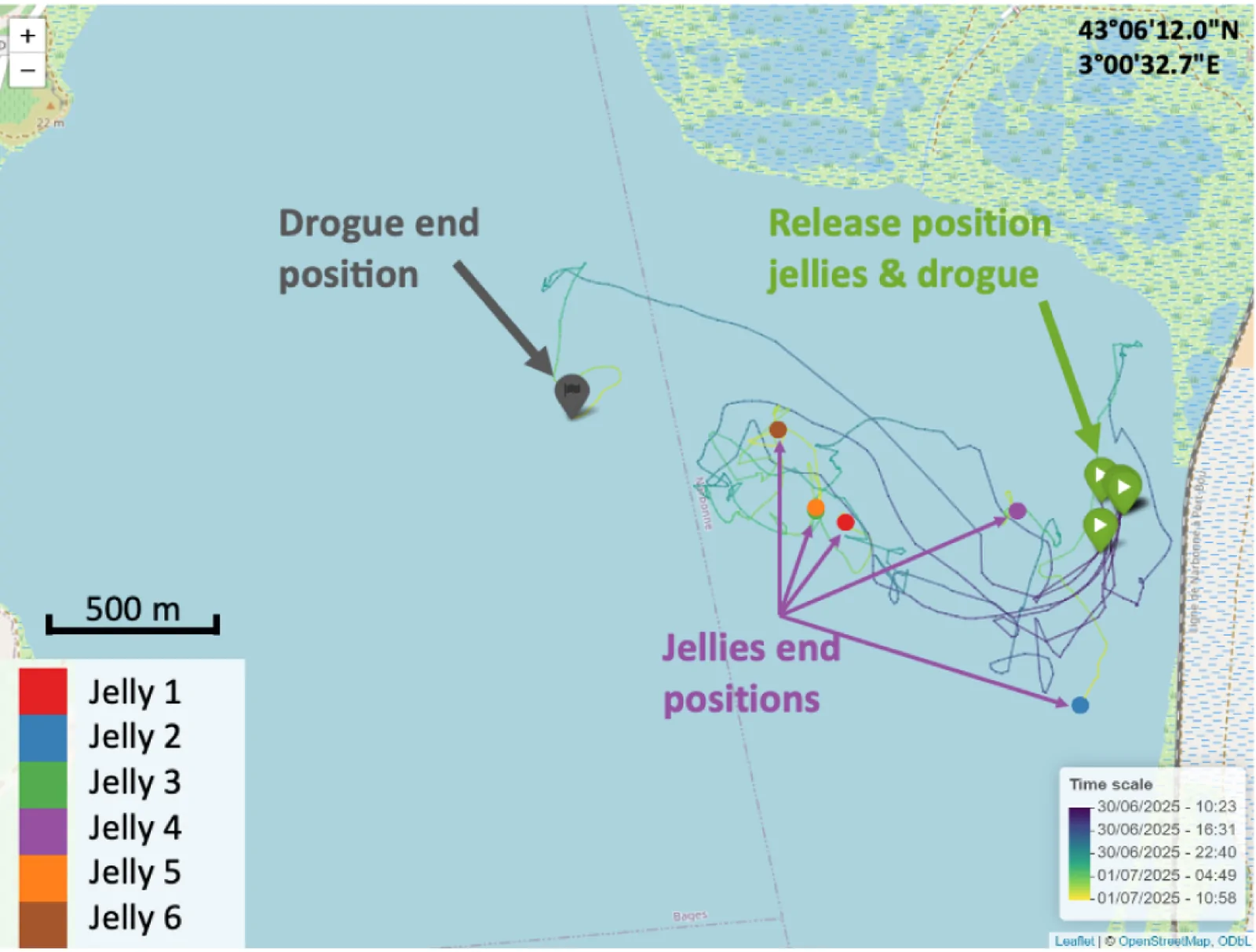
Trajectories from a 24-h deployment, six tagged jellyfish and a drogue have been released at the same point (green point with a triangle). Color dots are the recovery locations.

This workflow was used during actual jellyfish deployments and not only during bench or static technical tests. The received data were sufficient to monitor active tags during field operations, support recovery actions, and reconstruct post-deployment trajectories for tagged jellyfish and control drogues.

One main concern regarding biologging applications is to avoid negative impact of the device on the animal behavior. In our case study, two analysis have been conducted regarding this subject. The first approach considered was a theoretical mechanical study. For this study, we have calculated the energy needed to pull a half-immerged sphere of the size of the tag. This needed energy has been compared to literature data giving the energy used by a jellyfish to swim. Pulling the tag induces less than 1% of additional energy which we assume to be small enough to consider a limited impact. The second approach was a statistical analysis of swimming speed and pulse frequency of tagged and untagged jellyfish. For this study, we simultaneously filmed tagged and untagged jellyfish using a drone. Trajectories have been tracked. No statistical difference has been found for speed and pulse frequency between tagged and untagged jellyfish. This limited impact is mainly due to the fact that the tag is a sphere of 4.5 cm diameter, whereas the tagged jellyfish were between 25 and 35 cm long. These analyses corroborate observations of tagged jellyfish swimming with ease.

Fig. 13 presents the trajectories of six jellyfish and a drogue over 24 h. First observation is the fact that jellyfish movement does not consist only in drifting. There is significant contribution of the voluntary swimming activity. Extended statistical analysis conducted on the whole deployments brought additional information. Indeed for current speed over 4.09 cm s^−1^, jellyfish actively swim against the current. In addition, the stronger is the current, the stronger the jellyfish swim against it.

### 7.3 Performance characterization

**LoRaWAN reception in the lagoon.** Reception performance was assessed both during routine deployments and through dedicated zone-based tests in the lagoon. Since the final field design retained the nitinol LoRa antenna integrated into the lid, only the reception results obtained with this configuration are reported here.

The antenna and network configurations were evaluated during a controlled field test conducted in July 2024 at 16 stations distributed throughout the lagoon. At each station, the devices were put at the surface of water and maintained stationary for approximately 10 min. They were configured to wake periodically, allow up to approximately 30 s for GNSS acquisition, and transmit a LoRaWAN uplink at a nominal interval of approximately 90 s. In practice, this protocol produced four to five complete acquisition and transmission events per device at each station.

For clarity, a simplified five-zone reception figure was retained in the manuscript. In these tests, the TTN configuration with the nitinol antenna yielded reception rates between 50% and 100%, depending on the zone. Four of the five reported zones showed reception rates between 83% and 100%. Overall, reception was spatially variable across the lagoon, but remained generally satisfactory in the main deployment areas. Local performance depended on gateway placement and site geometry (see Fig. 14).

Across the five campaigns of 2025, 7198 onboard cycle records were retained, of which 5042 had a corresponding message in the TTN records. The resulting pooled reception rate was 70%. Campaign-level pooled reception ranged from 62.6% to 77.5%.

**Fig. 14:**
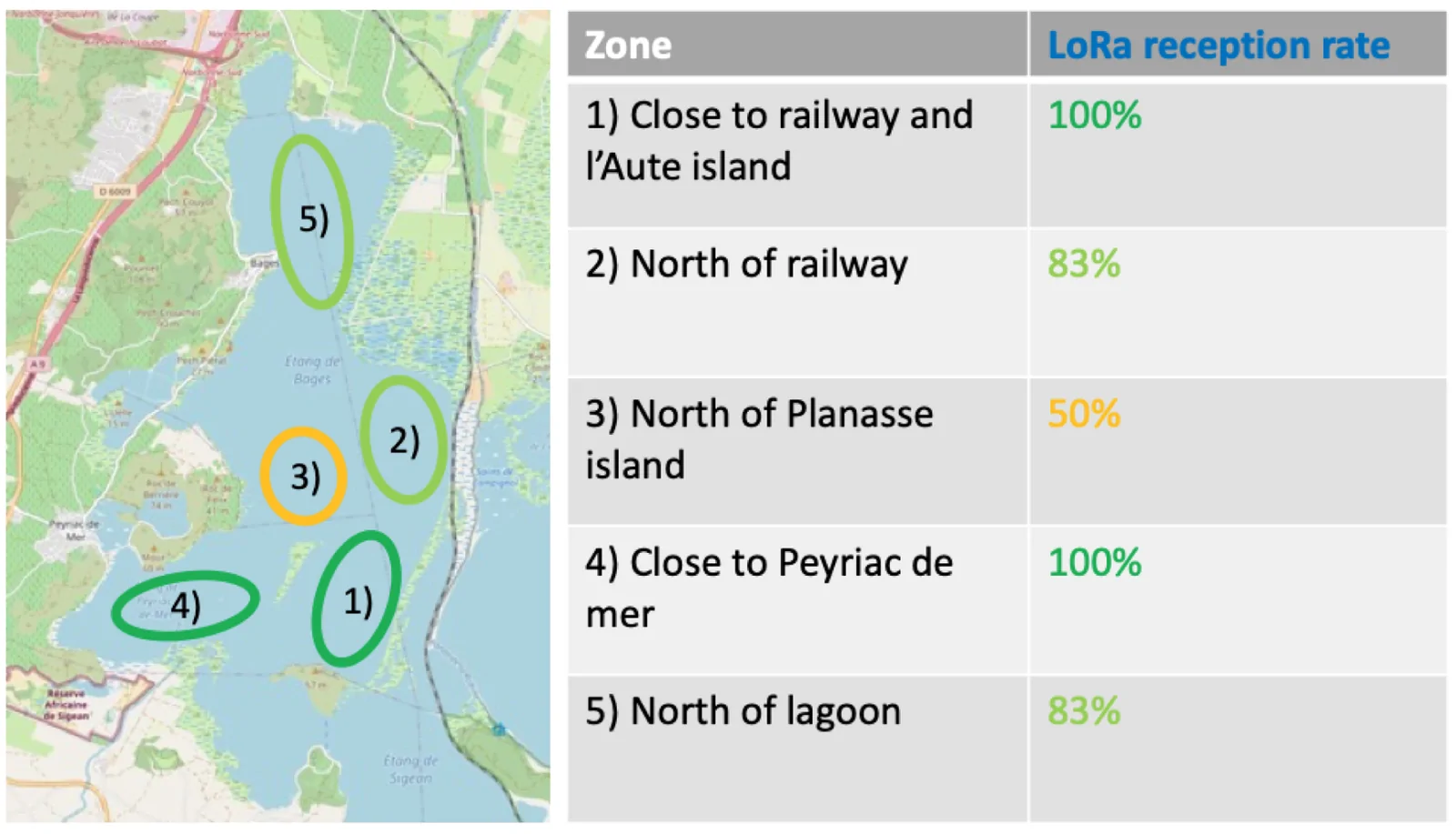
Reception rates over the Bages-Sigean lagoon using the three LoRaWan gateways deployed on the shore of the lagoon.

**GNSS operation.** The GNSS subsystem used in the float was previously characterized in a dedicated study on frugal floating devices based on the same hardware family and application context [13]. That previous study showed that fix quality and 2D/3D status were more informative than EHPE alone for selecting reliable fixes, that an embedded fix-selection strategy reduced GNSS-related energy use by about 40%, and that the corresponding theoretical autonomy increased from about 9 to 15 days under the tested scenario without hardware modification.

This prior characterization mainly supports the practical GNSS strategy used in the float. In operational lagoon conditions, the device was able to repeatedly acquire usable positions while remaining compatible with a frugal power budget.

One main objective of location transmission is the recovery of the device. In 2024, 7 over the 13 deployed devices (53.8%) were recovered. The embedded algorithm for GNSS location improvement has been implemented for 2025 deployments, resulting in 26 recoveries out of 30 deployments (86.7%). The total recovery rate is 76.6% (33/43).

**Mission duration and robustness.** In-the-field deployments showed that the hardware supported a wide range of deployment durations:

• 4 short deployments (4 in 2024, 0 in 2025) of about 1 h under fast duty-cycle settings (typically 1 or 2 min),
• 26 intermediate deployments (4 in 2024, 22 in 2025) covering day–night cycles (<30h) under intermediate duty-cycle settings (typically 5 or 10 min),
• 13 multi-day deployments (5 in 2024, 8 in 2025) up to 96 h under slower duty-cycle settings (typically 15 min).

The enclosure also showed practical robustness for field use. In internal testing, the assembled float remained watertight during a 72 h immersion test at 1.5 m depth. Although the device is intended as a surface float rather than a submerged logger, this result provides a useful robustness margin against splash exposure, handling, and temporary immersion. In complementary, no waterproof failure has been reported during the deployments.

**Data continuity and fallback operation.** A practical strength of the system is that it does not rely exclusively on continuous network availability. When reception is available, the system supports live tracking, field supervision, and recovery assistance. When reception is degraded or temporarily unavailable, onboard memory provides a fallback route for later data extraction after physical recovery of the tag. In practice, the hardware combines live tracking when coverage is available with recovery-oriented logging when coverage is incomplete.

### 7.4 Capabilities and limitations

**Capabilities**

• Periodic onboard GNSS acquisition with low-power duty-cycled operation.
• Live transmission of compact tracking packets through a standard LoRaWAN infrastructure.
• End-to-end workflow demonstrated from floating tag to TTN, MQTT export, storage, and operator notifications.
• Field operation demonstrated over deployment durations ranging from about 1 h to 96 h.
• Recovery-oriented design combining live monitoring with onboard memory backup.
• Lightweight floating architecture adapted to jellyfish tracking through a surface relay approach.

**Limitations**

• Radio reception depends on local gateway placement and is not spatially uniform across the study area.
• The system provides surface positions of the float rather than a full three-dimensional trajectory of the animal.
• Position acquisition frequency and mission duration remain constrained by GNSS activity and battery capacity.
• As the GNSS ceramic antenna is inside the casing for reliability reason, the GNSS fix acquisition can only be guaranteed when the tag is at the surface. Indeed as salted water dramatically attenuates RF signal, it can avoid correct functioning of the GNSS receiver, if it goes over the antenna.
• Continuous real-time reception cannot be guaranteed in all field areas.

## CRediT authorship contribution statement

**Quentin Ponzo:** Writing – original draft, Validation, Software, Methodology, Data curation. **Andrea Sauviat:** Validation, Software. **Delphine Bonnet:** Supervision, Project administration, Funding acquisition, Conceptualization. **Vincent Kerzerho:** Writing – review & editing, Supervision, Methodology, Funding acquisition, Data curation, Conceptualization.

## Ethics declaration

This study was conducted in accordance with the following guidelines for animal welfare and/or reporting: Directive 2010/63/EU on the protection of animals used for scientific purposes. Ethics committee approval was not required. Cnidarians are completely excluded from Directive 2010/63/EU on the protection of animals used for scientific purposes.

## Declaration of generative AI and AI-assisted technologies in the manuscript preparation process

During the preparation of this work, the authors used ChatGPT (OpenAI) to improve the generation of tables in LaTex and the language of the manuscript. After using this tool, the authors reviewed and edited the content as needed and take full responsibility for the content of the publication.

## Declaration of competing interest

The authors declare the following financial interests/personal relationships which may be considered as potential competing interests: Delphine Bonnet reports financial support was provided by French National Research Agency. If there are other authors, they declare that they have no known competing financial interests or personal relationships that could have appeared to influence the work reported in this paper.
